# Variation of Anthocyanin, Phenol, and Antioxidant Capacity in Straw among Rice Varieties and Growing Locations as a Potential Source of Natural Bioactive Compounds

**DOI:** 10.3390/plants11212903

**Published:** 2022-10-28

**Authors:** Suchada Jumrus, Supapohn Yamuangmorn, Jeeraporn Veeradittakit, Suthaphat Kamthai, Sithisavet Lordkaew, Teewara Suwan, Sansanee Jamjod, Chanakan Prom-u-thai

**Affiliations:** 1 Agronomy Division, Department of Plant and Soil Sciences, Faculty of Agriculture, Chiang Mai University, Chiang Mai 50200, Thailand; 2 Lanna Rice Research Center, Chiang Mai University, Chiang Mai 50200, Thailand; 3 Division of Packaging Technology, School of Agro-industry, Faculty of Agro-industry, Chiang Mai University, Chiang Mai 50200, Thailand; 4 Center of Agricultural Resource Systems, Faculty of Agriculture, Chiang Mai University, Chiang Mai 50200, Thailand; 5 Center of Excellence in Natural Disaster Management (CENDIM), Department of Civil Engineering, Faculty of Engineering, Chiang Mai University, Chiang Mai 50200, Thailand

**Keywords:** agricultural waste, rice straw, natural bioactive compounds, environmentally friendly, waste management

## Abstract

This study evaluated the variation in bioactive compounds (anthocyanins, phenols, and antioxidants) among 22 rice varieties in the same growing locations and among four varieties collected from eight different provinces in Northern Thailand. Wide variation in anthocyanins, phenols, and antioxidant capacity was established, ranging from 1.6 to 33.0 mg/100 g, 249.9 to 477.7 mg gallic acid/100 g, and 0 to 3,288.5 mg trolox equivalent/100 g, respectively. The highest straw anthocyanin and phenol concentrations were found in KDK (a traditional photoperiod-sensitive variety with purple pericarp and leaves) and K4 (an advanced, photoperiod-insensitive variety with purple pericarp and leaves), while the highest antioxidant capacity was found in KH CMU (an improved traditional photoperiod sensitive variety with a purple pericarp and green leaves) and K4. The variation of the bioactive compounds was also found in the same variety grown at different locations, e.g., the KDMl105 grown in Prayao province had a straw anthocyanin concentration higher than when grown in Mae Hong Son province. The effect was also observed in phenol content and antioxidant capacity when the same rice variety was grown across various locations. A significant correlation between total phenol and antioxidant capacity was observed across rice varieties and growing locations but was not found between anthocyanin and antioxidant capacity. This study found that the bioactive compounds in rice straw varied among rice varieties and growing locations. Straw phenol acts as a major antioxidant that can be used as a characteristic for the selection of rice varieties with high antioxidant capacity for use at the industrial scale for the processing of food, pharmaceuticals, and medicinal products.

## 1. Introduction

Globally, the valuable food molecule with a large number of bioactive compounds that potentially benefit human health by playing an important role in preventing illnesses has been focused on both plant and animal sources [1]. This includes large sources of natural biologically active compounds for application on a large scale in the industrial sectors, e.g., in food, supplements, pharmaceuticals, and medicines [2,3]. At the industrial scale, sources of material such as agricultural waste products (including rice straw) produced on a small scale are normally only used for animal feed, and a large amount is left in the field. The unused byproducts of rice straw in the field are generally burnt, resulting in economic waste and environmental air pollution [2,4,5], especially in areas where rice crops are widely cultivated. A more efficient way of use may help to add value and decrease the disposal of these by-products that can become a serious problem for the environment in many ways [6,7]. The opportunity of using the by-products from brewer’s spent grain provided from black beer production as a fortifying ingredient in bakery products was reported to potentially increase nutritional values and volatile compounds that can benefit human health [8].

In Asia, the rice crop is grown and consumed as the main source of energy among the populace; the Food and Agriculture Organization of the United Nations (FAO) estimates that approximately 740 million tons of rice are harvested worldwide, of which 90% (approximately 670 million tons) is produced and consumed in Asia, including Thailand, Vietnam, the Philippines, Indonesia, India, and Bangladesh [9]. It has been calculated based on the harvest index that every kilogram of rice grain is accompanied by the production of 1.0–1.5 kg of straw that is disposed of as waste each year [7,10]. Recent studies have reported that these byproducts contain bioactive compounds such as amino acids, phenolic acids, flavonoids, vitamin E, γ-oryzanol, and pigmented compounds that have potential benefits for human health [11,12]. Thus, rice straw can be a source of natural biologically active materials for processing in the industrial sector. However, limited information is available concerning the stability of the bioactive compounds in rice straw after rice crop harvesting regarding rice varieties and growing locations.

Several research studies have reported that rice straw contains a variety of bioactive compounds. Karimi et al. [13] were among the first to identify the bioactive compounds in rice straw varieties using high-performance liquid chromatography (HPLC); they found that rice straw contained phenolic compounds such as gallic acid, caffeic acid, pyrogallol, and salicylic acid as well as flavonoids such as kaempferol, apigenin, rutin, daidzein, genistein, and myricetin. Rice straw contains cellulose, hemicellulose, and lignin as well as sugars such as glucose, galactose, mannose, xylose, and arabinose [14,15] and a significant amount of hexose and pentose representing a potential source of bio-ethanol production [15]. In addition, rice straw can be used for producing L-lactic acid by *Lactobacillus casei* [16], as well as a natural sweetener, xylitol, that is widely used in industries such as food preparation, and pharmaceutical products by *Candida Subtropicalis* WF79*,* a microbe using xylose from rice straw [17]. However, the variation in the contents of bioactive compounds in rice straw among different varieties and growing sites have not yet been examined. We hypothesized that there is wide variation in the contents of the compounds among the varieties, and that this may depend on several environmental factors in the growing conditions. Therefore, this study was carried out to evaluate the variation of bioactive compounds among different rice varieties grown in the same conditions between growing sites and varieties. The information would be very useful for the selection of rice variety and growing location for the utilization of rice straw.

## 2. Results

### 2.1. Experiment 1: Variation of Bioactive Compounds in Rice Straw among 22 Rice Varieties

**Figure 1:**
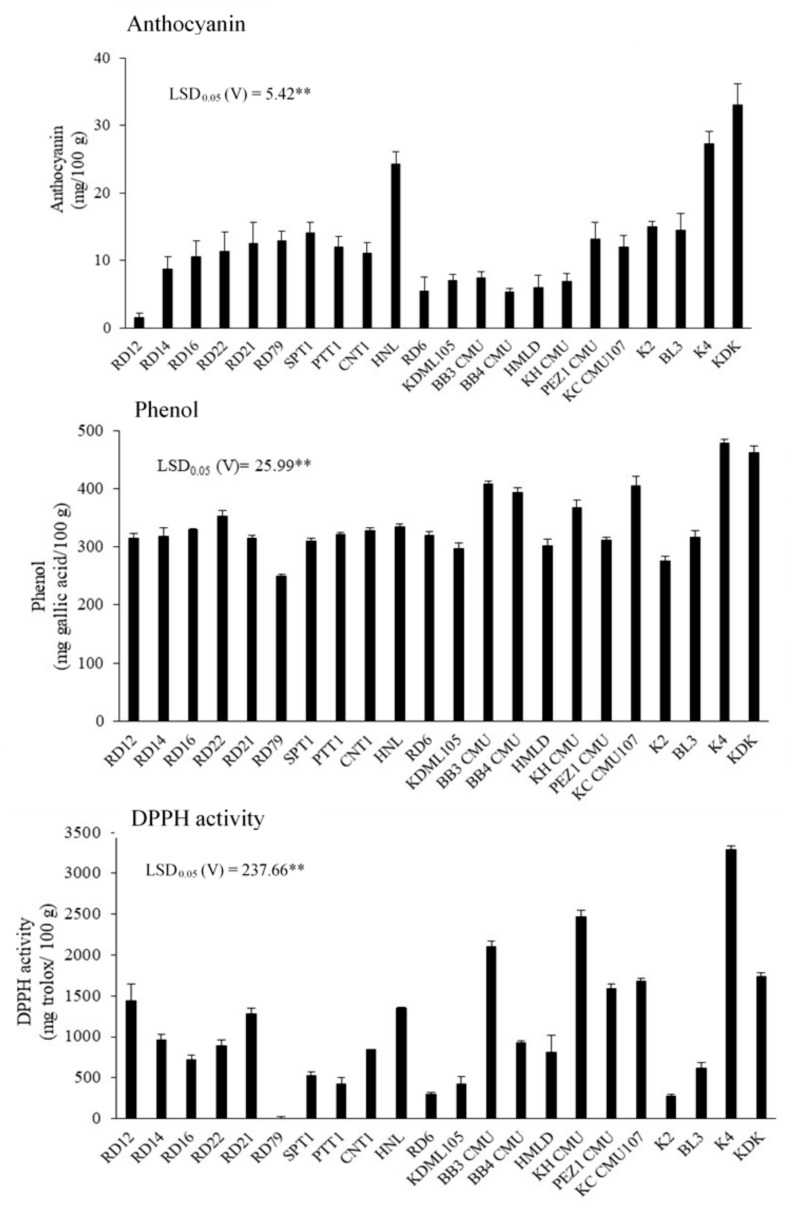
Concentrations of anthocyanin and phenol and DPPH activity in rice straw among 22 Thai rice varieties grown in the same conditions. The error bars above the graphs indicate SE (*n*= 5). ** indicates a significant difference by least significant difference (LSD) tests between rice varieties at *p* < 0.01.

Wide variation in the contents of the bioactive compounds in the rice straw was found among the 22 rice varieties, especially for the anthocyanin concentration and DPPH activity (*p* < 0.05) (Figure 1). The anthocyanin concentration in rice straw ranged from 1.6 to 33.0 mg/100 g. The highest straw anthocyanin concentration was found among the three purple caryopsis varieties of HNL, K4, and KDK and was the highest in the KDK variety. The three varieties each have a purple pericarp; K4 and KDK have purple leaves, but HNL has green leaves. A narrower range of concentration was found for phenols, ranging from 249.9 to 477.7 mg gallic acid/100 g (*p* < 0.05). The highest concentrations were found in the two varieties of K4 and KDK with purple pericarp and leaves. The antioxidant capacity determined by the DPPH activity ranged from 0 to3288.5 mg trolox equivalent/100 g. The highest concentrations were found in KH CMU and KDK. KDK has a purple pericarp and purple leaves, while KH CMU has a purple pericarp and green leaves. Some varieties had very low antioxidant capacity, for example RD79 that has a colorless pericarp and green leaves.

**Figure 2:**
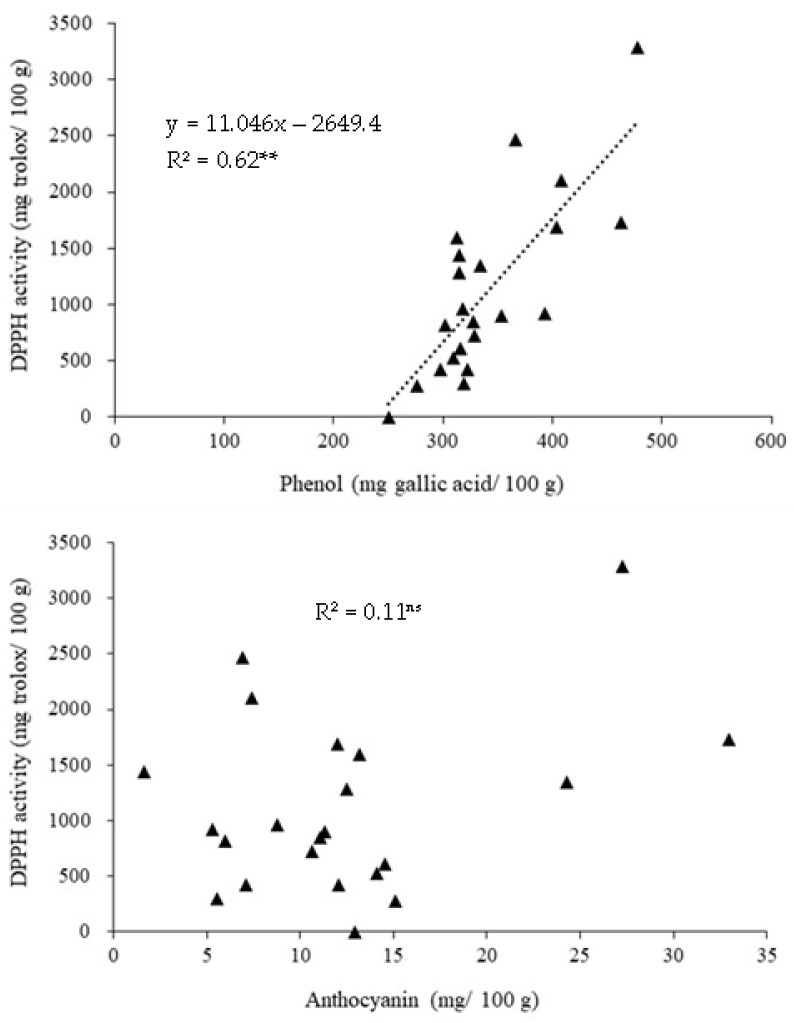
The relationship between phenol and DPPH activity and between DPPH activity and anthocyanin concentration in rice straw among 22 Thai rice varieties. **, ^ns^ indicate a significant difference and no significant difference at *p* < 0.05, respectively.

There was a significant relationship between the straw phenol concentration and DPPH activity among the 22 rice varieties (R^2^ = 0.62, *p* < 0.05), but such a relationship was not found between straw anthocyanin concentration and DPPH activity (R^2^ = 0.11, *p* > 0.05) (Figure 2).

The phenolic acid profile was analyzed in rice straw comparing the two varieties, KDK, purple leaves variety and KDML105, green leaves variety (Table 1). Gallic acid was found to be the dominant compound in rice straws of both varieties, a higher concentration of 398.01 mg/100 g was found in KDK compared with 131.56 mg/100 g in KDML105. The higher concentration of epicatechin, quercetin and *o*-coumeric acid were also found in KDK compared with KDML105, while about the same concentration was found in catechin, epigallocatechin gallate. On the other hand, *p*-coumeric acid and rosmarinic acid were detected in KDK rice straw, but not in KDML105.

### 2.2. Experiment 2: Variation of Bioactive Compounds in Rice Straw among Eight Locations in Northern Thailand

**Figure 3:**
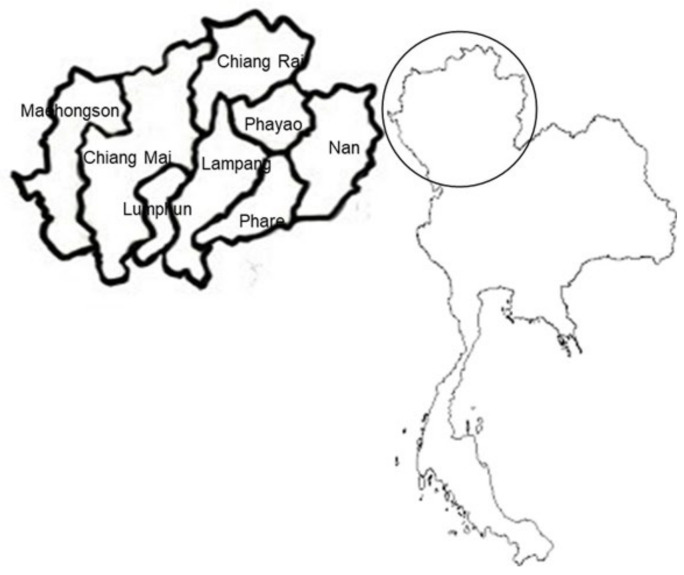
The details of eight provinces in Northern Thailand where the samples of rice straw were collected to determine the concentration of bioactive compounds.

The rice straw samples were collected from eight provinces in Northern Thailand, where diverse soil properties occur, and different varieties of rice are grown in various provinces (Figure 3). The soil texture varied among loam, loamy sand, loamy clay, and loamy clay with sand. The soil pH varied from 5.04 to 6.41. There were 1.56–2.94% organic matter, 0.08–0.15% total N, 2.09–29.94 mg/kg available P (Bray II) and 26.32–253.8 mg/kg extractable K (Table 2).

**Figure 4:**
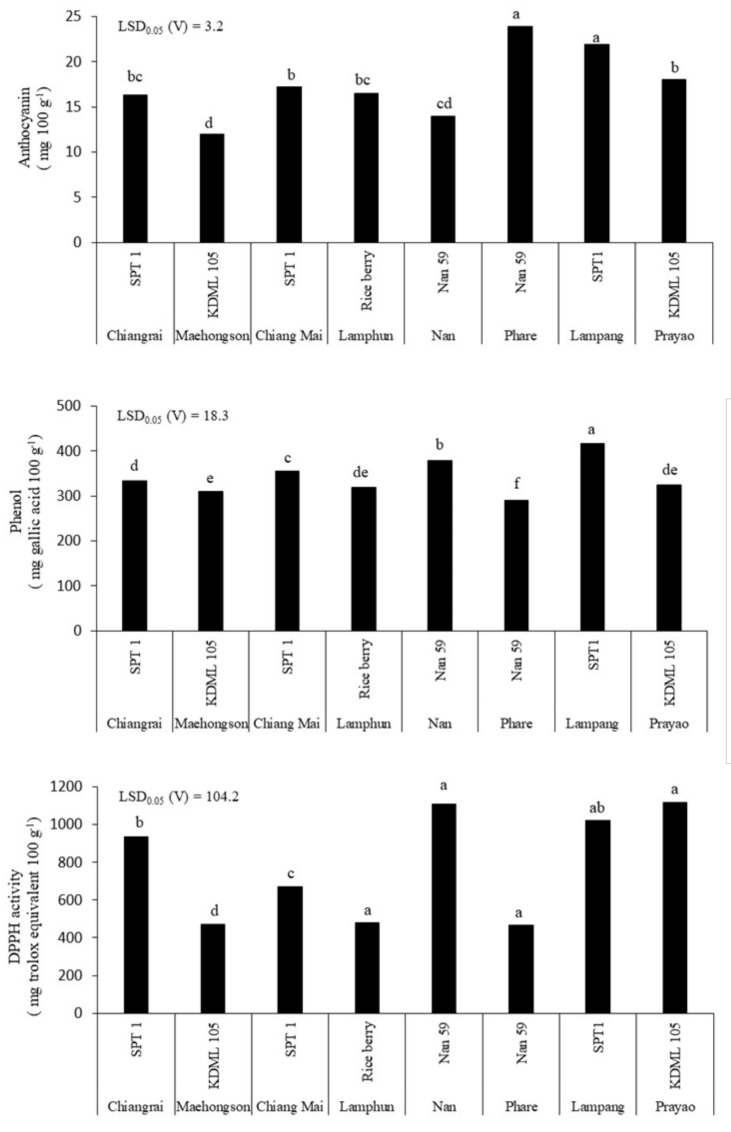
Concentrations of anthocyanin and phenol and DPPH activity in rice straw among eight Thai rice varieties grown in eight provinces in Northern Thailand. The letters above bars indicate a significant difference by least significant difference (LSD) between rice varieties at *p* < 0.01.

Wide variation in anthocyanin and phenol contents and DPPH activity in rice straw was found across the varieties and within the same variety grown in different locations (*p* < 0.05) (Figure 4). The straw anthocyanin concentration ranged from 11.9 to 23.9 mg/100 g, with the highest concentrations being found in Nan59 grown in Phare province and SPT1 grown in Lampang province. The lowest concentration was found in KDML105 grown in Mae Hong Son province. Within the same variety, KDML105 grown in Prayao province had a higher straw anthocyanin concentration than when grown in Mae Hong Son, while SPT1 grown in Lampang had a higher straw anthocyanin concentration than when grown in Chiang Rai or Mae Hong Son provinces. The straw phenol concentration varied from 290.6 to 417.4 mg gallic acid/100 g. The highest straw phenol concentration was found in SPT1 grown in Lampang province, while the lowest was Nan59 grown in Phare province. A similar straw phenol concentration average of 318.1 mg gallic acid/100 g was found in KDML105 grown between Mae Hong Son and Prayao provinces, while SPT1 grown in Mae Hong Son (310.8 mg gallic acid/100 g) had a lesser concentration than grown at Chiang Mai (354.7 mg gallic acid/100 g) and Chiang Rai 9 (334.5 mg gallic acid/100 g), respectively. Nan59 grown in Nan had a higher straw phenol concentration (359.6 mg gallic acid/100 g) than when grown in Phare (290.6 mg gallic acid/100 g). The straw DDPH activity fluctuated among the varieties and growing locations, ranging from 465.1 to 1118.3 mg trolox equivalent/100 g. The highest DPPH activities were observed in KDML105 grown in Prayao, SPT1 grown in Lampang, and Nan59 grown in Nan provinces, while the lowest DPPH activity was found in Nan59 grown in Phrae, Rice berry grown in Lamphun, and KDML105 grown in Mae Hong Son provinces. Among KDML105 grown at different locations, straw DPPH activity was about two times higher than in Prayao (1118.3 mg trolox equivalent/100 g) compared with those grown in Mae Hong Son province (472.0 mg trolox equivalent/100 g). SPT1 grown in Lampang (1020.8 mg trolox equivalent/100 g) had a similar concentration as that from Chiang Rai (937.0 mg trolox equivalent/100 g), about two times higher than when grown in Chiang Mai province (670.8 mg trolox equivalent/100 g).

**Figure 5:**
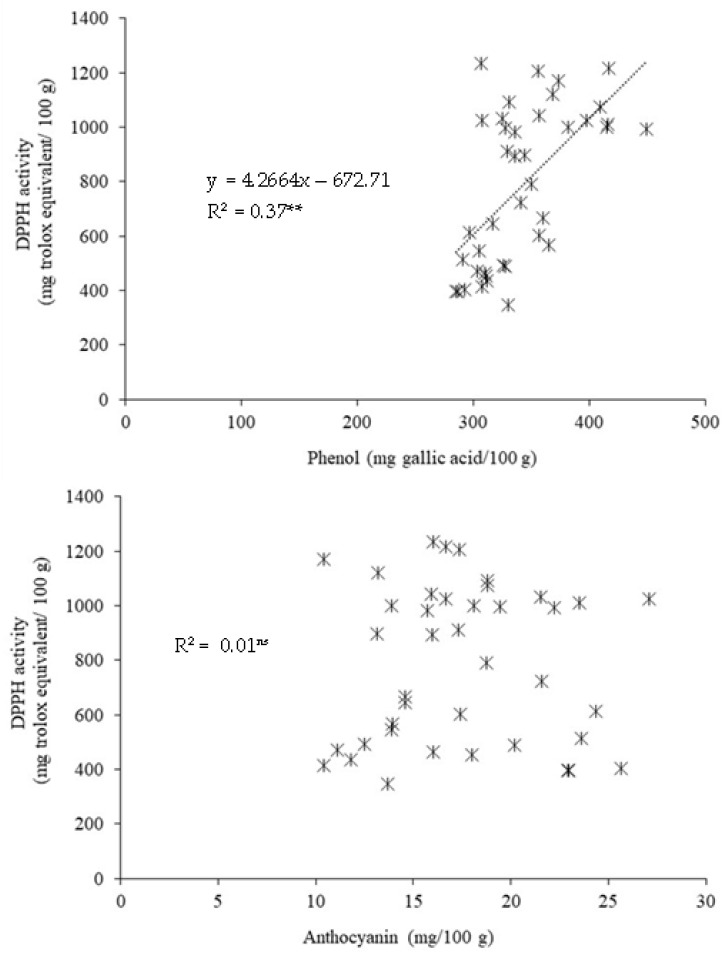
The relationship between phenol and DPPH activity and between DPPH activity and anthocyanin concentration in rice straw among eight Thai rice varieties grown in different provinces in northern Thailand. **, ^ns^ indicate a significant difference and no significant difference at *p* < 0.05, respectively.

There was a positive relationship between straw phenol and DPPH activity among rice varieties grown in different provinces (R^2^ = 0.37, *p* < 0.05), but such a relationship was not found between straw anthocyanin and DPPH activity (R^2^ = 0.01, *p* > 0.05) (Figure 5).

## 3. Materials and Methods

This study comprised two main experiments as detailed below.

### 3.1. Experiment 1: Variation of Bioactive Compounds in Rice Straw among 22 Rice Varieties

The field experiment was arranged in a completely randomized design (CRD) with three independent replications. A total of 22 rice varieties were grown in the wet season from June-December 2020 at the research station field of Chiang Mai University, Thailand. The details of each rice variety are given in Table 3. Seedlings were prepared by germinating rice seeds for 25–30 days. The seedlings were manually transplanted to a single plant per hill, with spacing between hills of 25 × 25 cm in 3 × 3.5 m^2^ plots per treatment replication. N-P-K fertilizer with a 15-15-15 formulation was applied at 125 kg ha^−1^ one day before transplanting. The N fertilizer of 46-0-0 was applied at 125 kg ha^−1^ at tillering stage and 62 kg ha^−1^ at the heading stage, similar to farmers’ practical management. Each plot was flooded with water to a depth of 10 cm throughout crop growth and drained 14 days before harvesting. The samples of rice straw were collected at the harvesting time before freeze-drying. The dried samples were ground with a hammer mill and stored in a freezer at −25 ° C before being used in the laboratory.

#### Chemical Analysis

Anthocyanin was analyzed independently from each replication by the modified pH-differential method of Abdel-Aal et al. [18]. About 2.5 g of freeze-dried ground rice straw sample was extracted with 24 mL of acidified methanol (70% methanol and 30% 1.5 N HCl, *v*/*v*) with shaking for 1 h, and then the sample was filtered through Whatman No. 1 filter paper. The supernatant was collected and added to the two buffer solutions 0.025 M potassium chloride buffer pH 1.0 and 0.4 M sodium acetate buffer pH 4.5. The absorbance of anthocyanin was measured at 520 and 700 nm using a spectrophotometer (Biochrom, Model Libra S22, Harvard Bioscience, Inc., London, England. The absorbance of the anthocyanin pigment was expressed as cyaniding 3-glucoside, and the main anthocyanin in rice straw was calculated as follows:

Anthocyanin = A × MW x DF × 1000/ε × L(1)

where A = (A520 nm–A700 nm) pH 1.0—(A520 nm–A700 nm) pH 4.5 MW = 449.2 g mol^−1^ for molecular weight of cyanidin 3-glucoside

DF = the dilution factor,

ε = 26,900 molar absorbance, and

L = 1 cm for cell path length

For phenol analysis, aliquots (2 g) of straw rice sample were extracted with three changes of 20 mL 50% methanol for 60 min each time. The extraction mixture was centrifuged at 3500× *g* for 5 min each time, and the supernatants were pooled for analysis of total extractable phenol using the Folin Ciocalteu method [19].

Antioxidant capacity was determined by the free radical scavenging activity of 2, 2-diphenyl-1-picrylhydrazyl (DPPH). The DPPH scavenging activity percentage of the absorbance of DPPH was calculated by plotting against each quantity of the extraction to produce a regression line. Trolox (0.4 mM) in methanol was used as a standard to convert the inhibition capability of the samples to the trolox-equivalent antioxidant activity. The ratio of the slopes of the regression lines of the extract solution and the trolox solution was defined as the trolox equivalent antioxidant capacity. This was then converted to µmol trolox equivalents per g rice straw.

The phenolic acid profile was analyzed in rice straw by extracting the samples with ethanol/water (95:5, *v*/*v*) according to the method of Arribas et al. [20]. The ethanolic fraction of the extracts was evaporated under reduced pressure (40 °C) in order to obtain a residue by using a rotary evaporator (R-300, Buchi, Japan). Then, the phenolic compounds were analyzed by applying the method of Mighri et al. [21]. Analytical liquid chromatography was performed using an Agilent 1260 Infinity II series chromatogragh, coupled with an Agilent 6130 electrospray ionization quadrupole mass spectrometer (Agilent Tech., Santa Clara, CA, USA). Separation was executed using an Ultra C18 column (5µm 4.6 × 250 mm; Restek, Bellefonte, PA, USA). The mobile phase was composed of A (0.2% acetic acid in 95% water and 5% MeOH) and B (0.2% acetic acid in 50% water and 50% acetonitrile) with a linear elution gradient: 0–45 min, 10–20% B; 45–85 min, 20–55% B; 85– 97 min, 55–100% B; 97–110 min, 100% B; the initial conditions were held for 10 min as a re-equilibration step. The flow rate of the mobile phase was 0.5 mL/min, the column temperature was maintained at 40 °C, and the injection volume was 20 µL. Spectra were obtained in the negative selected ion monitoring mode and processed using OpenLab software. High-purity nitrogen was used as a nebulizer and auxiliary gas. The mass spectrometer was operated in negative ion mode with a capillary voltage of −3.5 V, a nebulizing gas flow of 1.5 L/min, a dry gas flow rate of 12 L/min, a dissolving line temperature of 250 °C, a voltage detector of 1.35 V and the full scan spectra from 100 to 1200 m/z with 250 ms/spectrum

### 3.2. Experiment 2: Variation of Bioactive Compounds in Rice Straw among Eight Locations in Northern Thailand

#### 3.2.1. Sample Collection

The samples of rice straw and soil where the straw rice was cultivated were collected from eight different provinces in Northern Thailand. The soil samples were analyzed, and the details of soil profiles and rice varieties collected in the field are listed in Table 2. The samples of rice straw were randomly collected in 10 spots for each replication with a total of five independent replications from a representative variety widely grown in each province. The collected samples were freeze-dried and stored in the freezer at −25 °C before being used for bioactive compounds analysis as explained above.

#### 3.2.2. Statistical Analysis

Analysis of variance (ANOVA) was conducted to detect differences in the concentrations of anthocyanin and phenol and DPPH activity in rice straw among rice varieties and location sites using Statistic 9 (analytical software SX). Data were tested for normality by the normal probability plot method before being analyzed as factorials in a CRD. The least significant difference (LSD) test at *p* < 0.05 was applied to compare the means for significant differences between rice varieties and location sites. Pearson correlation analysis was used to test the associations between each pair variables.

## 4. Discussion

This study has established that rice straw contains bioactive compounds, even during crop maturity after the rice is harvested from the field. Wide variation in the contents of bioactive compounds was observed regarding the concentrations of anthocyanins and phenols as well as DPPH activity among rice varieties grown in the same locations and under the same management, indicating genotypic variation among the varieties. Significant variation was also found within the same variety grown in different locations with a diverse range of soil fertility profiles, management practices, and other environmental factors such as temperature and light duration and intensity, indicating the interaction effect between rice variety and growing conditions on the accumulation of straw bioactive compounds. Thus, the selection of rice varieties with the appropriate growing conditions would achieve optimal contents of bioactive compounds that could be used for the benefit of human health.

Previous studies reported that rice straw is a lignocellulosic material that contains 32–47% cellulose, 19–27% hemicellulose, and 5–24% lignin [22]. The release of phenolic acid from the bound lignin form showed high antioxidant activity that could be used in the food, pharmaceutical, and medicinal industries [23,24,25]. The bioactive compounds in rice straw as natural materials have rarely been investigated, especially among the varieties with purple leaves. The current study confirmed the presence of bioactive compounds in rice straw after crop maturity and harvesting, especially among the varieties with purple leaves and pericarp. The highest straw anthocyanin and phenol concentrations were found in K4 and KDK (Figure 1), illustrating the correlation between anthocyanin synthesis and/or accumulation in the source (leaves) and sink (grain) parts that should be further investigated via in-depth studies. Anthocyanins are bioactive compounds with high nutritional value in plants that have antioxidant and anti-inflammatory properties and thereby can reduce the risk of serious chronic diseases such as cancers and diabetes [26,27]. A previous study reported that the variation in anthocyanin concentration among purple rice varieties (purple coloration in the leaves) ranged from 170 to 210 mg/100 g in leaf blades and from 67 to 100 mg/100 g in stem + leaf sheath, and the concentrations declined with plant age in all four varieties [28]. The current study found that straw anthocyanin concentrations ranged from 1.6 to 33.0 mg/100 g among the 22 varieties grown in the same conditions, amounts that were much lower than in the young fresh leaves of purple rice, although using young leaves may affect the yield, as leaves are the major source of plant photosynthesis. Although the straw bioactive compounds in rice varieties were present at lower concentrations in comparison to fresh leaves and rice kernels [28,29], the use of these compounds not only provides benefits to human health but also ameliorates the environmental problem of air pollution from burning straw in the field.

Additionally, the current study observed a variety × growing location interaction in the variation of straw bioactive compounds. A previous study reported that the cultivation practices could be varied depending on environmental conditions such as light and temperature; all these factors could affect the synthesis of bioactive compounds such as anthocyanins and phenolic acids [29,30,31]. However, the optimum environmental conditions for maximizing the concentrations of compounds in rice straw have rarely been studied. For rice grains, a previous study showed that growing purple rice under flooded soil conditions resulted in higher anthocyanin accumulation, and a strong effect was found in upland rice [32]. These results indicated that anthocyanin synthesis responds to growing conditions in different water regimes, although straw anthocyanin was not measured when growing rice plants under similar conditions. Thus, other environmental factors, e.g., soil fertility and nutrient management, also affect the bioactive compounds in rice straw. The current study found that straw bioactive compounds varied within the same rice variety grown in different locations, e.g., KDML105 had an anthocyanin concentration and antioxidant capacity higher than those grown at Prayao compared with Mae Hong Son province. The variation of compounds within the same variety across locations was also found in SPT1 and Nan59. This confirmed that growing conditions at each location influenced bioactive compound accumulation in rice straw depending on rice varieties, and further distinguished the effect of each factor. Yamuangmorn et al. [28] reported that applying nitrogen fertilizer increased anthocyanin in the leaf blade and stem + leaf sheath among purple rice varieties. In a recent report, the addition of mineral elements, especially calcium, strongly increased grain anthocyanin concentration by three-fold compared to the control, while selenium effectively increased anthocyanin in the leaves [33]. Rice grain anthocyanin was induced by applying ZnO nanoparticles at 200 mg/L and was accompanied by an increase in enzyme antioxidant activity [34]. This study indicated that there was no association between the straw bioactive compounds and soil fertility profile, although there was a slight negative correlation between straw anthocyanin and soil phosphorus concentration. Although there are some studies reporting the enhancement of anthocyanin via manipulation of environmental factors, the responses of different experiments have been varied. Therefore, the study of interaction effects between rice variety and growing conditions on anthocyanin accumulation in purple rice will provide the necessary information for the selection of rice variety and the appropriate growing conditions to maintain a high anthocyanin content. For example, the selection of rice straw with purple leaf would result in higher straw anthocyanin concentration than green leaf varieties as indicated in this study, but it may vary according to the growing condition. Thus, the stability of straw anthocyanin may relate to many factors such as rice variety, temperature and humidity as was previously reported [29] which could be the reason for no correlation between straw anthocyanin concentration and antioxidant capacity in this study. To confirm the relationship, more rice varieties are required to include in future study.

The correlation between straw total phenol and antioxidant capacity in both experiments indicate that phenol can be a key compound that acts as an antioxidant in rice straw. Phenolic compounds are secondary metabolites found in plants that act as scavenge free radicals, reducing oxidative stress and protecting cells from possible risk, while in humans, their antioxidant characteristics are responsible for the prevention of chronic diseases such as obesity, diabetes, atherosclerosis, cancer, and cardiovascular disorders [35]. Additionally, phenolic acid profile analysis in the current study has shown that gallic acid was the major compound playing a key role in antioxidative properties in rice straw in both purple and green leaves varieties, though about three times the concentration was higher in the purple leaves compared with green leaves. Moreover, *p*-coumeric acid and rosmarinic acid were found in the straw of purple leave, but not in the green leave variety. Thus, these could be one of the reasons supporting the higher antioxidant activity in the straw of purple leave varieties.

The selection of rice varieties with high antioxidant capacity can use total phenol as an indicator for the future of rice straw as a natural source of bioactive compounds in the industry. This should be of benefit to farmers and increase their income, and the community will benefit from the decreased risk of respiratory diseases caused by air pollution from the burning of straw in the field.

## 5. Conclusions

Rice straw is a waste material from the production of rice crops; less than 10% is used for animal feed, soil mulching, and mushroom production. Most rice straw is left in the field and burned for the next crop, causing air pollution and thereby impacting human health. Adding value to rice straw by extracting its remaining natural bioactive compounds after the harvesting process is not only beneficial to human health but also environmentally friendly. This study establishes that the bioactive compounds remain in dry rice straws even after the rice is harvested from the field, but a wide variation of the compound in anthocyanins, phenols, and antioxidant capacity can be found among different rice varieties and growing locations. This indicates that the selection of rice varieties, especially among the purple leaf varieties grown in the appropriate locations and management, can affect the bioactive properties of rice straw. The variation in bioactive compounds remains to be further evaluated as to the effects of environmental factors; this would help to maximize the benefits of bioactive compounds in rice straw. Additionally, a significant correlation between total phenol and antioxidant capacity across rice varieties and growing locations in this study indicates that the straw phenol acts as a major antioxidant in rice straw as it is not found the correlation between anthocyanin and antioxidant capacity. The phenolic acid analysis has revealed that gallic acid is the major phenolic acid substance in rice straw playing a major role in anti-oxidative properties and a higher concentration was found in the purple than the green leaf varieties. Therefore, the straw phenol concentration is suggested as a characteristic for the selection of rice varieties with high antioxidant capacity for use at the industrial scale for the processing of food, pharmaceuticals, and medicinal products. Thus, this value-added to rice straw is not only the strategy for environmentally friendly by reducing pollution from burning rice straw in the field, but it would also help to increase farmers’ income by selling rice straw, the by-product of rice cultivation, to the industrial sectors for processing of functional food and other purposes. However, future research is needed to clarify in more detail the function of the natural bioactive compounds in rice straw.

## Figures and Tables

**Table 1:** Phenolic acid concentration in rice straw of two selected varieties (KDK and KDML105) grown in the same conditions.

Phenolic Acid	Concentration (mg/100 g)	
	KDK	KDML105
Gallic acid	398.01 ± 0.55	131.56 ± 0.11
Catechin	12.99 ± 0.03	12.64 ± 0.07
Epicatechin	101.48 ± 8.79	50.63 ± 18.42
Gallocatechin gallate	nd	nd
Epigallocatechin gallate	26.70 ± 0.41	20.78 ± 4.18
Caffeic acid	37.37 ± 0.17	48.79 ± 0.03
Naringin	nd	nd
*p*-coumeric acid	20.69 ± 0.49	nd
Rosmarinic acid	17.13 ± 0.27	nd
Quercetin	43.79 ± 1.36	36.17 ± 2.50
*o*-coumeric acid	28.63 ± 2.34	17.29 ± 0.42

All values are mean ± SE (*n* = 3); nd, not detectable.

**Table 2:** Details of soil chemical profile and name of rice variety grown in eight provinces in northern Thailand in experiment 2.

Province	Soil Texture	Rice Variety	pH	Organic Matter (%)	Total N (%)	Available P (mg/kg)	Extractable K (mg/kg)
Chiangrai	Loamy sand	SPT1	6.41	2.94	0.14	25.77	253.8
Mae Hong Son	Loamy sand	KDML105	5.49	1.56	0.08	29.94	42.86
Chiang Mai	Loam	SPT1	5.07	1.84	0.10	15.93	37.92
Lamphun	Loam	Rice berry	5.72	1.67	0.11	72.42	118.2
Phare	Loamy sand	Nan59	6.06	2.46	0.13	20.98	168.34
Nan	Loamy-clay	Nan59	5.24	1.82	0.08	2.09	26.32
Lampang	Loam	SPT1	5.65	2.34	0.11	9.92	119.2
Prayao	Loamy-clay-sand	KDML105	5.04	2.36	0.15	4.61	35.46

The values are mean from 2 replications. Soil chemical property was determined by soil pH (1:1, soil:water), organic matter (Walkley–Black method); total N (Kjeldahl method); available phosphorus (Bray II), exchangeable potassium (NH4OAc, pH 7).

**Table 3:** Details of rice varieties used to evaluate bioactive compound contents in straw in experiment 1.

No.	Variety	Symbol	Straw Color	Photoperiod	Ecotype	Endosperm Type
1	Rice Department 12	RD12	Straw	Modern improved Photoperiod insensitivity	Wetland	Glutinous
2	Rice Department 14	RD14	Straw	Modern improved Photoperiod insensitivity	Wetland	Glutinous
3	Rice Department 16	RD16	Straw	Modern improved Photoperiod insensitivity	Wetland	Glutinous
4	Rice Department 22	RD22	Straw	Modern improved Photoperiod insensitivity	Wetland	Glutinous
5	Rice Department 21	RD21	Straw	Modern improved Photoperiod insensitivity	Wetland	Non-glutinous
6	Rice Department 79	RD79	Straw	Modern improved Photoperiod insensitivity	Wetland	Non-glutinous
7	San Patong 1	SPT1	Straw	Modern improved Photoperiod insensitivity	Wetland	Glutinous
8	Pathum Thani 1	PTT1	Straw	Modern improved Photoperiod insensitivity	Wetland	Non-glutinous
9	Chai Nat 1	CNT1	Straw	Modern improved Photoperiod insensitivity	Wetland	Non-glutinous
10	Hom Nil	HNL	straw	Modern improved Photoperiod insensitivity	Wetland	Non-glutinous
11	Rice Department 6	RD6	Straw	Traditional improved Photoperiod sensitivity	Wetland	Glutinous
12	Khao Dawk Mali 105	KDML105	Straw	Traditional improved Photoperiod sensitivity	Wetland	Non-glutinous
13	Bue Bang 3 CMU	BB3 CMU	Straw	Traditional improved Photoperiod sensitivity	Upland	Non-glutinous
14	Bue Bang 4 CMU	BB4 CMU	Straw	Traditional improved Photoperiod sensitivity	Upland	Non-glutinous
15	Hom Mali Daeng	HMLD	Straw	Traditional improved Photoperiod sensitivity	Wetland	Non-glutinous
16	Kham Hom Morchor	KH CMU	Straw	Traditional improved Photoperiod sensitivity	Upland	Glutinous
17	Pi Ei Zu 1 CMU	PEZ1 CMU	Straw	Traditional improved Photoperiod sensitivity	Upland	Glutinous
18	Kum Chao Morchor107	KC CMU107	straw	Traditional improved Photoperiod sensitivity	Wetland	Non-glutinous
19	Advanced line K2	K2	Straw	Advanced line Photoperiod insensitivity	Wetland	Non-glutinous
20	Advanced line BL3	BL3	Straw	Advanced line Photoperiod insensitivity	Wetland	Non-glutinous
21	Advanced line K4	K4	purple	Advanced line Photoperiod insensitivity	Wetland	Non-glutinous
22	Kum Doi Saket	KDK	purple	Traditional improved Photoperiod sensitivity	Wetland	Glutinous

## Data Availability

Not applicable.

## References

[1] Salanță L.C., Uifălean A., Iuga C.-A., Tofană M., Cropotova J., Pop O.L., Pop C.R., Rotar M.A., Bautista-Ávila M., González C.V. (2020). Valuable Food Molecules with Potential Benefits for Human Health. The Health Benefits of Foods-Current Knowledge and Further Development.

[2] Peanparkdee M., Iwamoto S. (2019). Bioactive compounds from by-products of rice cultivation and rice processing: Extraction and application in the food and pharmaceutical industries. Trends Food Sci. Technol..

[3] Tanase C., Coșarcă S., Muntean D.L. (2019). A Critical Review of Phenolic Compounds Extracted from the Bark of Woody Vascular Plants and Their Potential Biological Activity. Molecules.

[4] Gadde B., Bonnet S., Menke C., Garivait S. (2009). Air pollutant emissions from rice straw open field burning in India, Thailand and the Philippines. Environ. Pollut..

[5] Toan N.S., Hanh D.H., Phuong N.T.D., ThiThuy P., Dong P.D., Gia N.T., Tam L.D., Thanh D.T.V., LokeShow K.S.K.P. (2022). Effects of burning rice straw residue on-field on soil organic carbon pools: Environment-friendly approach from a conventional rice paddy in central Viet Nam. Chemosphere.

[6] Daifullah A.A.M., Girgis B.S., Gad H.M.H. (2003). Utilization of agro-residues (rice husk) in small wastewater treatment plans. Mater. Lett..

[7] Adhikary S.K., Ashish D.K., Rudžionis Ž. (2022). A review on sustainable use of agricultural straw and husk biomass ashes: Transitioning towards low carbon economy. J. Sci. Total Environ..

[8] Farcas A.C., Socaci S.A., Tofana M., Muresan C., Mudura E., Salanta L., Scrob S. (2014). Nutritional properties and volatile profile of brewer’s spent grain supplemented bread. Romanian Biotechnol. Lett..

[9] The Food and Agricultural Organization (2020). Corporate Statistical database (FAOSTAT).

[10] Sarkar N., Ghosh S.K., Bannerjee S., Aikat K. (2012). Bioethanol production from agricultural wastes: An overview. Renew. Energy.

[11] Sfez S., Meester S.D., Dewulf J. (2017). Co-digestion of rice straw and cow dung to supply cooking fuel and fertilizers in rural India: Impact on human health, resource flows and climate change. J. Sci. Total Environ..

[12] Kumar A., Nayak A.K., Sharma S., Senapati A., Mitra D., Mohanty B., Prabhukarthikeyan S.R., Sabarinathan K.G., Mani I., Garhwal R.S. (2022). Recycling of rice straw—a sustainable approach for ensuring environmental quality and economic security: A review. Pedosphere.

[13] Karimi E., Mehrabanjoubani P., Keshavarzian M., Oskoueian E., Jaafar H.Z., Abdolzadeh A. (2014). Identification and quantification of phenolic and flavonoid components in straw and seed husk of some rice varieties (*Oryza sativa* L.) and their antioxidant properties. J. Sci. Food Agric..

[14] Belal B.E. (2013). Bioethanol production from rice straw residues. Braz. J. Microbiol..

[15] Tsunatu D.Y., Atiku K.G., Samuel T.T., Hamidu B.I., Dahutu D.I. (2017). Production of bioethanol from rice straw using yeast extracts peptone dextrose. Niger. J. Technol..

[16] Qi B., Yao R. (2007). L-Lactic acid production from Lactobacillus casei by solid-state fermentation using rice straw. BioResources.

[17] Liaw W.C., Chen C.S., Chang W.S., Chen K.P. (2008). Xylitol production from rice straw hemicellulose hydrolysate by polyacrylic hydrogel thin films with immobilized *Candida Subtropicalis* WF79. J. BioSci. Bioeng..

[18] Abdel-Aal el S.M., Young J.C., Rabalski I. (2006). Anthocyanin composition in black, blue, pink, purple, and red cereal grains. J. Agric. Food Chem..

[19] Folin O., Denis W. (1912). On phosphotungstic-phosphomolybdic compounds as color reagents. J. Biol. Chem..

[20] Arribas C., Pereira E., Barros L., Alves M.J., Calhelha R.C., Guillamón E., Pedrosa M.M., Ferreira L.C.F.R. (2019). Healthy novel gluten-free formulations based on beans, carob fruit and rice: Extrusion effect on organic acids, tocopherols, phenolic compounds and bioactivity. Food Chem..

[21] Mighri H., Akrout A., Bennour N., Eljeni H., Zammouri T., Neffati M. (2019). LC/MS method development for the determination of the phenolic compounds of Tunisian Ephedra alata hydro-methanolic extract and its fractions and evaluation of their antioxidant activities. South Afr. J. Bot..

[22] Binod P., Sindhu R., Singhania R.R., Vikram S., Devi L., Nagalakshmi S., Kurien N., Sukumaran R.K., Pandey A. (2010). Bioethanol production from rice straw: An overview. Bioresour. Technol..

[23] Jung K.A., Woo S.H., Lim S.R., Park J.M. (2015). Pyrolytic production of phenolic compounds from the lignin residues of bioethanol processes. Chem. Eng. J..

[24] Pouteau C., Dole P., Cathala B., Avérous L., Boquillon N. (2003). Antioxidant properties of lignin in polypropylene. Polym. Degrad. Stab..

[25] Garrote G., Cruz J.M., Moure A., Domınguez H., Parajó J.C. (2004). Antioxidant activity of byproducts from the hydrolytic processing of selected lignocellulosic materials. Trends Food Sci. Technol..

[26] Wang H., Liu D., Ji Y., Liu Y., Xu L., Guo Y. (2020). Dietary supplementation of black rice anthocyanin extract regulates cholesterol metabolism and improves gut *microbiota dysbiosis* in C57BL/6J mice fed a high-fat and cholesterol diet. Mol. Nutr. Food Res..

[27] Zheng H.X., Qi S.S., He J., Hu C.Y., Han H., Jiang H., Li X.S. (2020). Cyanidin-3-glucoside from black rice ameliorates diabetic nephropathy via reducing blood glucose, suppressing oxidative stress and inflammation, and regulating transforming growth factor β1/Smad expression. J. Agric. Food Chem..

[28] Yamuangmorn S., Dell B., Rerkasem B., Prom-u-thai C. (2018). Applying nitrogen fertilizer increased anthocyanin in vegetative shoots but not in grain of purple rice genotypes. J. Sci. Food Agric..

[29] Yamuangmorn S., Prom-u-Thai C. (2021). The Potential of High-Anthocyanin Purple Rice as a Functional Ingredient in Human Health. Antioxidants.

[30] Zaidi S.H.R., Zakari S.A., Zhao Q., Khan A.R., Shah J.M., Cheng F. (2019). Anthocyanin accumulation in black kernel mutant rice and its contribution to ROS detoxification in response to high temperature at the filling stage. Antioxidants.

[31] Yamuangmorn S., Laororng K., Saenchai C., Lordkaew S., Dell B., Prom-u-Thai C. (2022). Boron requirement for vegetative growth of Sacha inchi (*Plukentia volubilis* L.). J. Plant Nutr..

[32] Jaksomsak P., Rerkasem B., Prom-u-thai C. (2020). Variation in nutritional quality of pigmented rice varieties under different water regimes. Plant Prod. Sci..

[33] Bennett C., Sookwong P., Moolkam S., Mahatheeranont S. (2020). Effect of plant nutrients on anthocyanin content and 940 yield component of black glutinous rice plants. Int. J. Agric. Biol. Eng..

[34] Samart S., Chutipaijit S. (2019). Growth of pigmented rice (*Oryza sativa* L. cv. Riceberry) exposed to ZnO nanoparticles. Mater. Mater. Today: Proc..

[35] Okarter N., Liu C.S., Sorrells M.E., Liu R.H. (2010). Phytochemical content and antioxidant activity of six diverse varieties of whole wheat. Food Chem..

